# Factors that cause endodontic failures in general practices in Japan

**DOI:** 10.1186/s12903-018-0530-6

**Published:** 2018-04-27

**Authors:** Mikiyo Yamaguchi, Yuichiro Noiri, Yoshihiro Itoh, Shungo Komichi, Kyoko Yagi, Reo Uemura, Haruna Naruse, Saori Matsui, Nanako Kuriki, Mikako Hayashi, Shigeyuki Ebisu

**Affiliations:** Osaka University Graduate School of Dentistry, Osaka, Japan

**Keywords:** Refractory periapical periodontitis, Extraradicular biofilm, Endodontic failure

## Abstract

**Background:**

Bacterial biofilms that develop on root surfaces outside apical foramens have been found to be associated with refractory periapical periodontitis. However, several other factors cause endodontic failures apart from extraradicular biofilms. The aim of this study was to identify the factors causing endodontic failures in general practices in Japan.

**Methods:**

Patients diagnosed as having refractory periapical periodontitis by general practitioners and who requested endodontic treatment at Osaka University Dental Hospital were selected by checking medical records from April 2009 to March 2013. Factors causing endodontic failures were identified.

**Results:**

A total of 103 teeth were selected, and 76 teeth completed root-canal treatment. Tooth extractions were required for 18 teeth after or without endodontic treatment. Six teeth required apicoectomy after endodontic treatment. One tooth needed hemisection. One tooth needed intentional replantation. One tooth needed adhesion and replantation. The main causes of treatment failure were open apices (24 teeth), perforation (18 teeth), and root fracture (13 teeth). In six teeth with open apices that required apicoectomy or extraction, extraradicular biofilms may have been related to endodontic failure.

**Conclusions:**

Most endodontic cases diagnosed with refractory periapical periodontitis by general practitioners were compromised by any other factors rather than extraradicular biofilms.

**Electronic supplementary material:**

The online version of this article (10.1186/s12903-018-0530-6) contains supplementary material, which is available to authorized users.

## Background

It has been reported that the prevalence of healing after initial treatment and retreatment of root canals is 86% and 82%, respectively [1, 2]. Several factors, such as perforation and intraoperative complications, affect healing [1, 2].

The purpose of root-canal treatment (RCT) is thorough mechanical and chemical cleaning of an infected root-canal system, followed by its complete obturation with a filling material [3]. Removal of infected substances and avoidance of further intraoperative/postoperative infection are crucial for successful RCT. Failure of RCT can be because of infected substances inside the root canal or at extraradicular areas.

It has been reported that extraradicular infection associated with apical periodontitis does not respond well to RCT according to culture-based [4–8] and molecular studies [9–12]. Morphologic studies by our research team have shown that biofilms develop on root surfaces outside apical foramens of the teeth extracted for refractory periapical periodontitis and that gutta-percha points extrude in extraradicular areas removed during endodontic treatment for chronic periapical periodontitis [13]. Furthermore, bacterial DNA has been detected in extraradicular areas in 14 of 20 samples of refractory periapical periodontitis, and that bacterial species such as *Fusobacterium nucleatum*, *Porphyromonas gingivalis*, and *Tannerella forsythia* are detected frequently [14]. Our study excluded the possibility of contamination during sampling of apical periodontitis lesions for microbial analyses [14]. Furthermore, several case reports have revealed that extraradicular infection can be a cause of persistent symptoms [15, 16]. Therefore, the biofilm inhabiting extraradicular areas was one of the possible causes of refractory periapical periodontitis.

RCT tends to be blinded treatment and the root-canal system is morphologically complicated, thus several other factors can also cause the existence of infected substances and endodontic failures apart from extraradicular biofilms. In addition, in Japan, most patients requiring RCT are treated by general practitioners (GPs) and treatment is covered by health insurance. Therefore, setting aside sufficient time and using devices such as cone-beam computed tomography (CBCT) and dental operating microscopes (DOPs) that aid the chance of successful RCT for all patients is sometimes difficult.

We have observed endodontic failures diagnosed as refractory periapical periodontitis by GPs, who have requested us to conduct retreatments for such patients at Osaka University Dental Hospital (Osaka, Japan). In the treatment of such cases, several other factors causing endodontic failures other than extraradicular biofilms have been noted. The aim of the present study was to identify the factors causing endodontic failures in general practices in Japan.

## Methods

### Design and setting of the study

A retrospective observational study was undertaken in patients diagnosed with refractory periapical periodontitis by GPs who requested endodontic treatments at Osaka University Dental Hospital from April 2009 to March 2013. Patients in whom the factors causing endodontic failures had been obvious at the time they were introduced to Osaka University Dental Hospital were excluded.

### Data collection

Medical records of eligible patients were reviewed and the factors causing endodontic failures were identified based on the diagnoses and prognoses of re-treatments.

## Results

### Outcome

A total of 103 teeth met the inclusion criteria. Seventy-six teeth had completed RCT. Tooth extractions were required for 18 teeth after or without endodontic treatment. Six teeth needed apicoectomy after endodontic treatment. One tooth needed hemisection. One tooth needed intentional replantation. The other tooth needed adhesion and replantation. Distribution of the teeth treated in our study is given in Table 1.

**Table 1:** Distribution of the teeth treated

Locations	Total (n)	Completed RCT (n)	Apicoectomy (n)	Tooth extraction (n)	Hemisection (n)	Intentional replantation (n)	Adhesion and replantation (n)
Maxillary incisor	13	6	4	2	–	1	–
Maxillary premolar	18	12	2	2	–	–	1
Maxillary molar	26	21	–	5	–	–	–
Mandibular incisor	3	3	–	–	–	–	–
Mandibular premolar	12	12	–	1	–	–	–
Mandibular molar	31	22	–	8	1	–	–
Total	103	76	6	18	1	1	1

Completed RCT: Cases completed root canal treatment, Apicoectomy: Cases needed apicoectomy, Tooth extraction: Cases required tooth extraction, Hemisection: Cases needed hemisection, Intentional replantation: Cases needed intentional replantation, Adhesion and replantation: Cases needed adhesion and replantation

### Factors causing endodontic failure in patients who completed RCT

Main causes of treatment failure in patients who completed RCT were open apices (18/76), missed canal (12/76), insufficient enlargement of the root canal (10/76), perforation (9/76), fin isthmus (8/76), transportation (7/76), residual caries (7/76), root fracture (5/76), inaccessible root apex (4/76), and separated instruments (4/76) (Table 2). The number of cases using CBCT is given in Table 2.

**Table 2:** Factors causing endodontic failures in cases completed root canal treatments and the number of cases using CBCT

Factors	No. of Teeth	Percentage of Total	No. of cases using CBCT
Open apices	18	23.7	3
Missed canal	12	15.8	5
Insufficient enlargement of root canal	10	13.2	1
Perforation	9	11.8	2
Fin isthmus	8	10.5	3
Transportation	7	9.2	2
Residual caries	7	9.2	4
Root fracture	5	6.6	2
Inaccessible root apex	4	5.2	1
Separated instruments	4	5.2	1
Fenestration	2	2.6	2
Accessory canal	2	2.6	1
Endo-perio	2	2.6	1
Indefinite complaint	1	1.3	1
Periodontitis	1	1.3	0
Nonodontogenic toothache	1	1.3	0
Occlusal trauma	1	1.3	0
Total	76	100	20

Several factors were identified in one tooth

### Factors causing endodontic failure in patients requiring apicoectomy after endodontic treatment

The causes of treatment failure in patients who needed apicoectomy after endodontic treatment were open apices (3/6), fenestration (1/6), accessory canal (1/6), and transportation (1/6) (Table 3). The number of cases using CBCT is given in Table 3.

**Table 3:** Factors causing endodontic failures in cases needed apicoectomy and required tooth extractions and the number of cases using CBCT

Factors	No. of Teeth	Percentage of Total	No. of cases using CBCT
Apicoectomy			
Open apices	3	50	0
Fenestration	1	16.7	0
Accessory canal	1	16.7	1
Transportation	1	16.7	1
Total	6	100	2
Tooth extractions			
Perforation	8	44.4	1
Root fracture	7	38.9	3
Open apices	3	22.2	1
Inaccessible root apex	1	5.6	1
Separated instruments	1	5.6	1
Endo-perio	1	5.6	0
Total	18	100	6

Several factors were identified in one tooth in cases required tooth extractions

### Factors causing endodontic failure in patients requiring tooth extraction after or without endodontic treatment

The main causes of treatment failure in patients who required tooth extraction after or without endodontic treatment were perforation (8/18), root fracture (7/18), and open apices (3/18) (Table 3). The number of cases using CBCT is given in Table 3.

### Factors causing endodontic failure in patients requiring surgery after or without endodontic treatment

Combined endodontic and periodontic disease was the cause for treatment failure in the patient who needed hemisection. The cause of treatment failure in the patient who needed intentional replantation was perforation. The cause of treatment failure in the patient who needed adhesion and replantation was root fracture.

## Discussion

Success of endodontic treatment is dependent upon: (i) removal of bacteria from infected root canals; and (ii) control of secondary infection.

The inability to identify and access infected areas is a major cause of endodontic failure and disease persistence.

Recent developments in equipment such as CBCT and DOPs have improved the success rate of RCT. CBCT was developed in the late-1990s and overcomes the limitation of two-dimensional radiography to display three-dimensional structures of the root-canal system, especially in multi-rooted teeth [17, 18]. A recent retrospective cohort study showed that the prevalence of missed canals in endodontic-treated teeth and the effect of untreated canals on endodontic outcome could be evaluated using CBCT [19]. The prevalence of missed canals was 23.04% and teeth with a missed canal were 4.38-fold more likely to be associated with a lesion [19]. In the present study, in 12 of 76 patients who completed RCT, factors causing endodontic failure were missed canals and, in 5 of these 12 patients, CBCT was used to find missed canals. In patients who completed RCT by discovery of missed canals, some symptoms may have disappeared by thorough cleaning of discovered canals.

Furthermore, CBCT can be used to identify fin isthmus, accessory canals, and fenestration in three-dimensions. In the present study, fin isthmus, accessory canals, and fenestration were factors causing endodontic failure in 8, 2, and 2 of 76 patients who completed RCT, respectively. CBCT was used in 3 of 8 cases in whom the factor causing endodontic failure was fin isthmus, 1 of 2 cases in whom the factor causing endodontic failure was accessory canals, and in 2 of 2 cases in whom the factor causing endodontic failure was fenestration. In these cases using CBCT, identification using a three-dimensional image may have enabled access to infected fin isthmus and accessory canals to clean them. In patients who completed RCT but who had fenestration, the causes of tenderness of root apices became clear, and resulted in patients being agreeable to have a root-canal filling.

The DOP is also an important tool for endodontic treatments. It was introduced into endodontic treatments in the early-1990s. By providing enhanced lighting and visibility, DOPs aid: location of stenosed or hidden root canals obstructed by calcifications and reduced in size; repair of biologic and iatrogenic perforations; location of cracks and fractures; and removal of canal obstructions and separated instruments. Several studies have reported that DOPs improve significantly the ability to locate and negotiate canals [20–22]. For example, Baldassari-Cruz and colleagues showed that the number of second mesiobuccal (MB2) canals identified in maxillary molars increased from 51% with the naked eye to 82% with the DOP [20]. Other studies have shown that DOP use improves detection of MB2 canals to > 90% in maxillary first molars and 60% in maxillary second molars [21, 22]. In the present study, missed canal, perforation, root fracture and separated instruments were factors causing endodontic failure in 12, 9, 5, and 4 of 76 patients who completed RCT, respectively. A DOP may have enabled access to hidden root canals and locate perforations and fractures. Furthermore, A DOP can be used to access and remove infected dentin or separated instruments precisely and keep dentin loss to a minimum.

Understanding the three-dimensional structures of the root-canal system using CBCT and the DOP can prevent procedural accidents such as transportation.

In patients who completed RCT, some of the factors causing endodontic failures in patients who completed RCT could have been resolved by using CBCT and the DOP. Use of CBCT and the DOP is becoming common in general practices in Japan. However, the results of the present study have shown the difficulty of availability of such devices in general practices.

The most common factor causing endodontic failures in patients who completed RCT was open apices. In the apical root, there is constriction which, in general, is 0.5–1.5 mm coronal to the apical foramen, and is considered to be the part of the root canal with the smallest diameter. It is the reference point used most often as the apical termination for shaping, cleaning, and obturation. Resorption of apical roots associated with chronic apical periodontitis/trauma and over-instrumentation enlarge apical constriction, resulting in open apices. Because apical areas are irritated and violated mechanically during RCT by instruments or filling materials for teeth with open apices, symptoms such as vertical percussion pain and tenderness of apical areas or discomfort are not improved completely even though there is no obvious infected substance inside the root canal or at extraradicular areas. Therefore, such cases are diagnosed as refractory cases. In cases of resorption of apical roots or excessive apical enlargement, an appropriate working length shorter than the length of the root canal by 0.5–1 mm should be set. One can hypothesize that mechanical irritation for apical areas disappears and symptoms are relieved in patients who complete RCT but who have open apices by setting an appropriate working length.

In six patients with open apices requiring apicoectomy and tooth extraction after endodontic treatment, symptoms were not relieved by setting an appropriate working length and intra-canal cleaning. Extraradicular biofilms may have been associated with persistent symptoms in these patients. Previously we found, using morphologic and genetic analyses, that biofilms are formed in extraradicular areas in teeth with refractory periapical periodontitis, and proposed that extraradicular biofilms developing from the root canal via an apical foramen attach to the cementum around the root apex [13, 14]. Furthermore, we reported that the bacterial species detected from extraradicular biofilms were also detected from the root canal in the same teeth at a prevalence of 86.7% [14]. That result suggests that bacteria that remain in the root canal may become a source for extraradicular biofilms and open apices could encourage formation of extraradicular biofilms through the apical foramen.

Most cases of failed endodontic treatment by Japanese GPs were compromised by factors other than extraradicular biofilms. Therefore, to increase the chances of successful treatment, dissemination of educational endodontic programs with use of modern devices such as CBCT and DOPs for Japanese GPs and improvement in the environment to practice endodontic methods are desired.

Furthermore, regenerative endodontics (in which the aim is replacement of damaged pulp tissue with viable tissue) is attracting attention. It has been defined as “biologically-based procedures designed to physiologically replace damaged tooth structures, including dentin and root structures, as well as cells of the pulp-dentin complex” [23]. At present, the indication for regenerative endodontics is immature permanent teeth with pulpal necrosis. However, research into regenerative endodontics is being conducted internationally at many institutions and updated continuously [24]. Therefore, future expansion of cases in whom regenerative endodontics is indicated is promising. Regenerative endodontics may change the concept of RCT and decrease tooth loss due to apical periodontitis in the future.

## Conclusions

Most endodontic cases diagnosed with refractory periapical periodontitis by general practitioners were compromised by any other factors rather than extraradicular biofilms.

## Additional file

Additional file 1: Factors causing endodontic failures in all cases. (XLSX 115 kb)

## Abbreviations

CBCT: Cone-beam computed tomography
DOPs: Dental operating microscopes
GPs: General practitioners
MB2: Second mesiobuccal
RCT: Root-canal treatment
